# Two Cases of Israeli Spotted Fever with Purpura Fulminans, Sharon District, Israel

**DOI:** 10.3201/eid2405.171992

**Published:** 2018-05

**Authors:** Regev Cohen, Frida Babushkin, Maurice Shapiro, Martina Uda, Yafit Atiya-Nasagi, Dar Klein, Talya Finn

**Affiliations:** The Ruth and Bruce Rappaport Faculty of Medicine, Technion, Haifa, Israel (R. Cohen);; Laniado Hospital, Netanya, Israel (R. Cohen, F. Babushkin, M. Shapiro, M. Uda, T. Finn);; The Israel Institute for Biological Research, Ness-Ziona, Israel (Y. Atiya-Nasagi, D. Klein)

**Keywords:** Israeli spotting fever, Rickettsia conorii subsp. israelensis, rickettsia, case series, purpura fulminans, vector-borne infections, zoonoses, Sharon District, Israel

## Abstract

We report a series of 5 case-patients who had Israeli spotted fever, of whom 2 had purpura fulminans and died. Four case-patients were given a diagnosis on the basis of PCR of skin biopsy specimens 3–4 days after treatment with doxycycline; 1 case-patient was given a diagnosis on the basis of seroconversion. *Rickettsia* spp. from the 2 case-patients who died were sequenced and identified as *Rickettsia conorii* subsp. *israelensis.* Purpura fulminans has been described in association with *R. rickettsii* and *R. indica*, but rarely with *R. conorii* subsp. *israelensis.*

## Abstract 2

Genetic sequencing should be used to confirm cases because purpura fulminans is a rare finding.

*Rickettsia conorii* is the etiologic agent of Mediterranean spotted fever (MSF), which is considered one of the most severe and life-threatening rickettsial infections. Among the 4 strains of *R. conorii* (*R. conorii* subsp. *conorii*, *R. conorii* subsp. *caspia*, *R. conorii* subsp. *indica*, and *R. conorii* subsp. *israelensis*), *R. conorii* subsp. *israelensis*, which causes Israeli spotted fever (ISF), is believed to be the most virulent strain and shows a case-fatality rate of up to 32.3% in hospitalized patients (*1*). *R. conorii* is endemic to Israel (*2*). However, in recent years, *Rickettsia* spp. other than *R*. *conorii* have been identified and reported from Israel, including *R. africae*, *R. massiliae*, *R. sibirica* (*3**,**4*).

Within the *R. conorii* group, there are clinical and virulence differences. For example, infections with *R. conorii* subsp. *israelensis* have higher case-fatality rates than infections with the *R. conorii* Malish strain (29% vs. 13%), and *R. conorii* subsp. *israelensis* is rarely is associated with an eschar at the site of a tick bite (*5**,**6*). ISF has also been reported from other Mediterranean countries, including Portugal (*7*), Italy and Sicily (*6*), and Tunisia (*8*). *R. conorii* subsp. *conorii* that cause MSF are also endemic to Europe, Asia, and Africa.

ISF usually manifests as fever with a maculopapular rash, usually involving the palms of the hands and the soles of the feet. Patients who die from ISF typically have multiorgan failure, acute renal or hepatic failure, and acute encephalitis; all are attributed to the affinity of the *Rickettsia* spp. to endothelial cells with resultant vasculitis. The typical maculopapular rash might transform to become hemorrhagic with discrete purpurial lesions, but true manifestation of ISF purpura fulminans is not frequently reported in Israel or from other countries.

Because serologic cross-reactivity occurs across the spotted fever group (SFG) rickettsiae (*9*) and the primary means of diagnosis is through serum antibody assays, accurate distinction between different subspecies requires identification of the actual infecting bacterium. This cross-reactivity, overlap in geographic distributions, and the different clinical severities highlight the need to better differentiate between these rickettsial species. A correct diagnosis is critical for predicting the pathologic complications that would arise because of infection (*10*).

We report a case series of 5 patients with ISF from the same geographic area (Sharon District in Israel) (Tables 1, 2). Four of these case-patients were detected during April–May 2017. Two case-patients had purpura fulminans and both died. These cases were a part of a national outbreak of SFG rickettsiosis.

**Table 1:** Laboratory findings at hospitalization for 5 case-patients with Israeli spotted fever, Sharon District, Israel*

Parameter	Reference range	Case-patient 1	Case-patient 2	Case-patient 3	Case-patient 4	Case-patient 5
Leukocytes, cells/μL	4,000–11,000	10,700	7,500	5,000	7,700	8,600
PMNs, %	40–75	87	86	81	85	91
Platelets/μL	150,000–400,000	37,000	73,000	91,000	75,000	139,000
Sodium, mmol/L	136–145	126	123	130	133	127
Total bilirubin, mg/dL	0.3–1.4	2.5	0.4	0.9	0.7	0.8
AST, IU/L	0–32	106	184	167	65	69
ALT, IU/L	0–33	51	154	139	68	66
GGT, IU/L	0–40	50	103	62	26	106
LDH, IU/L	240–480	1044	875	1195	466	536
Creatinine, mg/dL	0.5–0.9	1.7	1.4	1.0	1.4	1.0
Creatine kinase, IU/L	39–190	298	1933	152	159	100
C-reactive protein, mg/L	0–5	122	350	164	88	240

*ALT, alanine aminotransferase; AST, aspartate aminotransferase; GGT, γ-glutamyl transferase; LDH, lactate dehydrogenase; PMN, polymorphonuclear leukocytes.

**Table 2:** Clinical characteristics of 5 case-patients with Israeli spotted fever, Sharon District, Israel*

Characteristic	Case-patient 1	Case-patient 2	Case-patient 3	Case-patient 4	Case-patient 5
Age, y/sex	75/F	51/F	38/ M	48/M	45/M
Concurrent condition	Hypertension	Mental retardation, diabetes mellitus	None	Alcoholism, HCV carrier, IVDU	Nephrolithiasis, psoriasis
Signs/symptoms at hospitalization					
Fever	Yes	Yes	Yes	Yes	Yes
Rash	Purpura fulminans	Maculopapular, then purpura fulminans	Maculopapular	Maculopapular	Maculopapular
Day of rash from disease onset	6	6	4	4	3
Hypotension	Yes	Yes	No	No	No
Multiorgan failure	Yes	Yes	No	No	No
Eschar	No	No	No	No	No
Headache	No	Unknown	Yes	No	No
Confusion	No	No	No	No	No
Myalgia	Yes	Unknown	Yes	No	No
Epidemiologic factor					
Animal exposure	Dog	Unknown	Dog	None	Dog
Tick exposure	No	No	No	No	No
Serologic result, day/result					
First sample	9/nonreactive	6/IgM titer >100, IgG titer borderline†	6/nonreactive	6/nonreactive	5/nonreactive
Second sample	19/IgM titer nonreactive, IgG titer 1:800†	NR	NR	NR	19/IgM titer >1:100, IgG titer 1:1,600†
PCR result, whole blood/doxycycline treatment day	NR	+/3	NR	NR	–/5
PCR result, skin biopsy specimen/doxycycline treatment day	+/4	+/3	+/4	+/3	–/3
Doxycycline treatment					
Day of illness	6	5	5	5	4
Day of hospitalization	0	2	0	1	0
Length of hospitalization, d	14	20	7	4	8
Outcome	Died	Died	Survived	Survived	Survived

*HCV, hepatitis C virus; IVDU, intravenous drug use; NR, not relevant/not obtained; +, positive; –, negative. †Results from the Israeli reference laboratory for rickettsial diseases.

## Case-Patient 1

**Figure 1:**
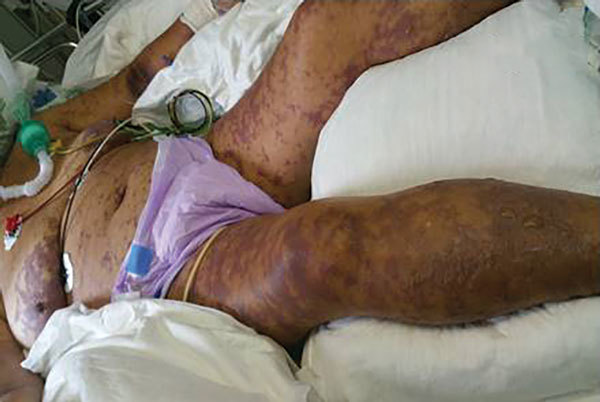
Case-patient 1, a 75-year-old woman with Israeli spotted fever and purpura fulminans, Sharon District, Israel, 3 days after hospitalization. Diffuse fern-leaf pattern of purpura and newly formed bulla on the legs are shown.

A 75-year-old woman with a history of hypertension, a tourist from Ukraine who had been staying with her daughter in Netanya for the previous 3 months, came to an emergency department in October 2016 with 6 days of fever, weakness, and anorexia. She had been exposed to 3 dogs at the house of her daughter but had no recollection of tick exposure. At admission, she was coherent but in a state of septic shock; she was hypotensive and had a disseminated, fern-leaf pattern, purpural rash over her body, including the face (Figure 1). Laboratory findings included leukocytosis, severe thrombocytopenia (23,000 platelets/μL), hyponatremia (sodium level 126 mmol/L), acute renal failure, increased levels of liver enzymes and creatine kinase (CK), and disseminated intravascular coagulation (Table 1).

**Figure 2:**
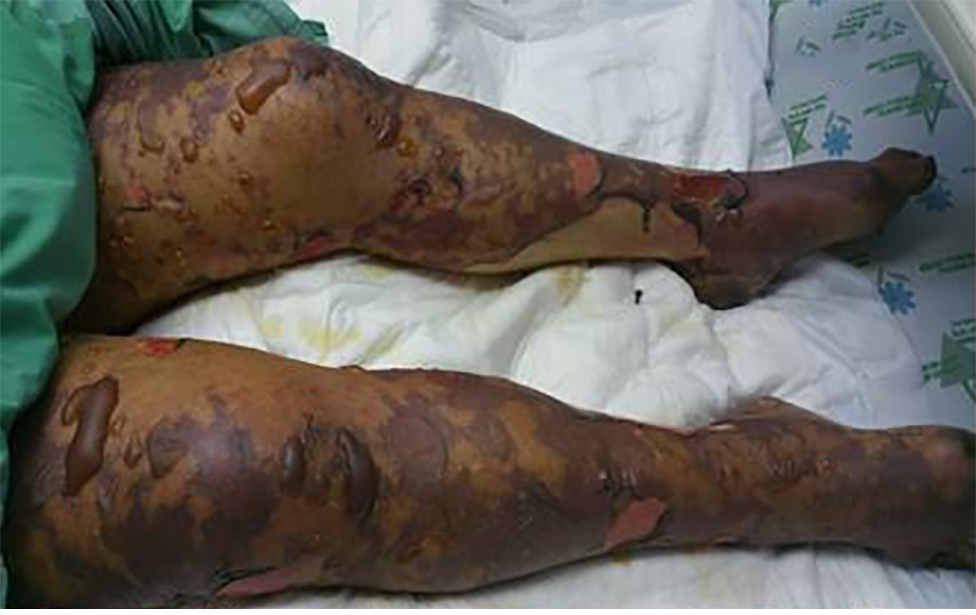
Case-patient 1, a 75-year-old woman with Israeli spotted fever and purpura fulminans, Sharon District, Israel, 7 days after hospitalization. A rash on the legs that had become bullous and contained clear serous fluid is shown.

She was admitted to the intensive care unit (ICU) and given broad-spectrum antimicrobial drug therapy, including intravenous doxycycline. During the next 2 days, multiorgan failure, severe jaundice, liver failure, and acute lung injury developed, and the rash became bullous with clear serous fluid (Figure 2). A skin biopsy specimen from a hemorrhagic lesion was obtained and tested by PCR. The result was positive for *R. conorii* subsp. *israelensis*. Serologic analysis for rickettsia at the time of admission (day 9 after disease onset) showed no reactivity, but another sample obtained 10 days later was positive (IgG titer 1:800) for SFG rickettsiae.

**Figure 3:**
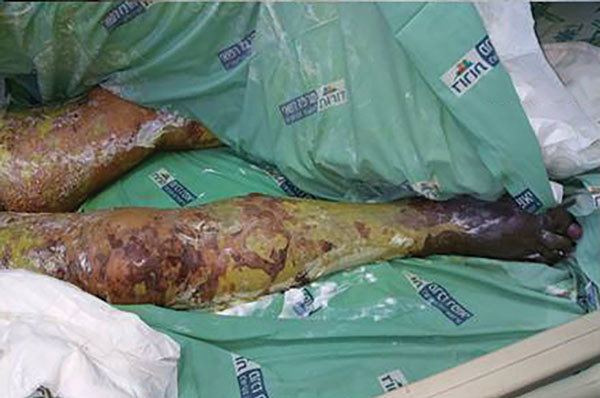
Case-patient 1, a 75-year-old woman with Israeli spotted fever and purpura fulminans, Sharon District, Israel, 14 days after hospitalization. Dusky skin lesions on the legs, necrosis at the extremities, and extensive desquamation are shown.

The skin lesions became dusky, necrosis appeared at the extremities (Figure 3), and there was extensive desquamation. The patient died of candidemia after 14 days in ICU. 

## Case-Patient 2

A 51-year-old woman with diabetes, who was mentally retarded and lived in a nursing home was hospitalized in an internal medicine ward on May 2017 with fever of no obvious source. She had no known exposure to dogs or ticks at the nursing home. The case-patient was hypotensive at admission, and laboratory results included a standard leukocyte count, thrombocytopenia (73,000 platelets/μL), hyponatremia, acute renal failure, increased levels of CK (1,933 IU/L) and liver enzymes, and a C-reactive protein level of 350 mg/L (Table 1). She was treated empirically with ceftriaxone.

On the third day of hospitalization, an infectious diseases consultation was performed, and intravenous doxycycline was added to the treatment regimen. She was transferred to the ICU unit, and the next day a maculopapular rash developed, which included the palms and soles; the rash rapidly became confluent and bullous, and skin necrosis followed.

PCR results for a whole blood sample and a skin biopsy specimen were positive for *R. conorii* subsp. *israelensis*. Serologic analysis on day 6 of the fever showed an IgM titer >1:100 and a borderline IgG titer of 1:100 for rickettsia. 

Despite treatment with doxycycline, multiorgan failure developed and the patient died of *Pseudomonas aeruginosa* bacteremia related to central line infection 3 weeks after admission.

## Case-Patients 3–5

Three other case-patients were admitted to Laniado Hospital (Netanya, Israel) during April–May 2017 for fever and a maculopapular rash involving the palms that appeared on the third or fourth day after fever onset. Two patients had exposure to dogs, but none reported exposure to ticks. One patient had alcoholism, but he had a benign illness course. The diagnosis for 2 patients was made by using PCR for skin biopsy specimens, and the diagnosis for the third patient was made by using seroconversion. Skin biopsy PCR results were positive despite 3–4 days of doxycycline treatment.

## Methods

### Serologic Analysis

Serologic diagnosis was made by using indirect immunofluorescence assays. Serum samples were obtained from 5 patients and tested for antibodies against *R. conorii* and *R. typhi* as described (*11*).

### DNA Extraction and PCR Detection

We extracted DNA from skin biopsy or whole blood specimens from 4 patients by using the QIAamp Mini Kit (QIAGEN, Valencia, CA, USA) according to the manufacturer’s instructions. We tested samples for SFG rickettsiae by using nested PCR to amplify a fragment of the 17-kDa protein antigen gene as described (*12*). For further identification, we performed an additional PCR by using primers derived from consensus sequences of the rickettsial outer membrane protein A gene (213F 5′-AATCAATATTGGAGCCGGTAA-3′ and 667R 5′-ATTTGCATCAATCGTATAAGTAGC-3′). We analyzed sequences by using BLAST (https://blast.ncbi.nlm.nih.gov/Blast.cgi). This fragment was identical to that of the Israeli tick typhus rickettsial outer membrane protein A gene (GenBank accession no. U83441.1).

## Discussion

SFG rickettsial disease is endemic to Israel and might be related to deaths of infected patients. However, manifestation of ISF as primary purpura fulminans has been described only rarely. Thus, we report patients with rickettsial disease and purpura fulminans.

Among various species of SFG rickettsiae, the types known to be associated with patient deaths are *R. rickettsii* (agent of Rocky Mountain spotted fever); *R. conorii* of the subtypes *conorii*, *israelensis*, and *indica*; and rarely *R. australis* (*13*). Although patient deaths are associated with vasculitis and multiorgan failure, purpura fulminans as part of the clinical presentation is a feature seen only for a small subset of the species, namely *R. rickettsii*, *R. australis* (*14*), and probably *R. conorii* subsp. *indica*. *R. conorii* subsp. *indica* was reported in several case series from the Indian subcontinent as a cause of fern-leaf pattern purpura, although the specific rickettsial subtype was infrequently identified in these studies (*15**–**20*).

Purpura fulminans is not a typical feature of ISF in reviews and in case series from Israel and other countries to which *R. conorii* subsp. *israelensis* is endemic (*5**,**6**,**21*). Although there are reports of deaths of patients in Israel who had a clinical picture suspected initially to be meningococcemia, fern-leaf purpura, as for 2 of our case-patients, was not part of the clinical descriptions (*22*). We have found only 1 case report of *R. conorii* subsp. *israelensis* infection and purpura fulminans (*23*). We report 2 additional cases.

The type of severe skin damage in the 2 case-patients we report is not the natural history of fatal spotted fever cases because it has not been described in other patients dying of ISF. The severe and confluent skin lesions undoubtedly contributed to bloodstream infections, leading to death in both cases. Because the differential diagnosis of purpura fulminans in Israel usually includes microorganisms other than rickettsiae, this deadly, unreported, unique, and potentially misleading manifestation of ISF must be recognized and treated in a timely fashion.

The clinical presentation of case-patients in this case series was typical of ISF and compatible with other case series of ISF (Table 2) (*5**,**24*). The reasons why 2 of these patients died and the other 3 patients had relatively mild disease is not entirely clear. Both case-patients who died were older; had hypotension, purpura fulminans, multiorgan failure, and acute renal failure; and showed increased levels of CK. Delay in doxycycline administration was evident for case-patient 1 (day 6 after illness onset) because she was hospitalized at a later time. A delay in doxycycline administration during medical observation occurred for case-patient 2, although treatment was given on day 5 after disease onset, which was similar to the timing for the 3 case-patients who survived.

We sequenced rickettsiae isolated from the 2 case-patients who died; both were positive for *R. conorii* subsp. *israelensis*. However, we did not perform sequencing for isolates from the other 2 case-patients who were positive by PCR for the SFG rickettsiae. These findings suggest that disease severity might be related to the specific subtype, and not to host factors, but further research is required. Alcoholism has been reported a risk factor for fatal disease (*5*), but the patient in this case series who had alcoholism had a benign course of illness.

With regards to epidemiologic, clinical, and laboratory findings, as expected, none of our patients had an eschar or known exposure to ticks. One patient had diarrhea, but 2 patients had constipation. All 5 case-patients had rash and laboratory abnormalities typical of ISF (Table 1), as described (*24*).

As previously reported (*6**,**24*), serologic analysis was not useful in the diagnosis of ISF because results were nonreactive for all case-patients early in the disease course and positive for 2 case-patients tested during the convalescent period (19 days after disease onset). In contrast, whole blood and skin biopsy specimen PCRs were helpful in obtaining a diagnosis. PCR results for skin biopsy specimens were positive for 4 of the 5 case-patients even though these PCRs were performed for 4 patients 3–4 days after doxycycline therapy was begun. The 1 case-patient with a negative PCR result for a skin biopsy specimen also had no inflammatory changes by histologic analysis, Thus, the negative PCR result for this patient probably represented a sampling error and not a false-negative result.

PCR for whole blood was performed for 2 patients: case-patient 2 showed a positive result after 3 days of doxycycline therapy, and case-patient 5 showed a negative result after 5 days of doxycycline therapy. Reduced sensitivity of whole blood PCR after beginning antimicrobial drug therapy has been reported (*24*). For the 4 case-patients with positive PCR results for skin biopsy specimens, the pathology report did not indicate a diagnosis of rickettsiosis, describing either intravascular coagulation and no vasculitis for the 2 patients who died and perivascular minimal lymphocytic infiltration not consistent with rickettsiosis for the other 2 patients.

Our cluster was a part of an outbreak throughout Israel during the spring and summer of 2017. This outbreak included deaths of 2 healthy young persons. One of these persons was a 15-year-old boy from the Sharon District. During July 2017, the Israeli Ministry of Health reported on an increased incidence of spotted fever cases (11 cases during January–May 2017); the yearly average is 6 cases (*25*).

In summary, during the spring and summer of 2017, there was an increased incidence of spotted fever in Israel, causing the deaths of 4 patients. Three of these patients acquired the disease in the Sharon District, and 2 were seen in our facility. Two of the case-patients who died were confirmed by sequencing to be infected with *R. conorii* subsp. *israelensis*; both of these patients had purpura fulminans and multiorgan failure. Purpura fulminans should increase the suspicion of ISF, along with other better-described pathogens, such as *Neisseria meningitidis*, and diagnostic and therapeutic measures must be taken urgently.

PCR of skin biopsy specimens is useful in providing a rapid and accurate diagnosis, even if performed 3–4 days after beginning of treatment, and PCR of whole blood might also show positive results during antimicrobial drug treatment early in the disease course. Histopathologic results for skin lesions might be inaccurate and cause a delay in diagnosis or an erroneous diagnosis. Features of *R. conorii* subsp. *israelensis* should be further explored primarily by performing genetic sequencing of all isolates from cases of spotted fever diagnosed in Israel to distinguish it from other spotted fever rickettsiae or *R. conorii* subsp. *conorii* that might cause a less aggressive disease.
